# The UK-DALE dataset, domestic appliance-level electricity demand and whole-house demand from five UK homes

**DOI:** 10.1038/sdata.2015.7

**Published:** 2015-03-31

**Authors:** Jack Kelly, William Knottenbelt

**Affiliations:** 1 Department of Computing, Imperial College London, London, SW7 2RH, UK

**Keywords:** Scientific data, Electrical and electronic engineering, Sustainability, Energy

## Abstract

Many countries are rolling out smart electricity meters. These measure a home’s total power demand. However, research into consumer behaviour suggests that consumers are best able to improve their energy efficiency when provided with itemised, appliance-by-appliance consumption information. Energy disaggregation is a computational technique for estimating appliance-by-appliance energy consumption from a whole-house meter signal. To conduct research on disaggregation algorithms, researchers require data describing not just the aggregate demand per building but also the ‘ground truth’ demand of individual appliances. In this context, we present UK-DALE: an open-access dataset from the UK recording Domestic Appliance-Level Electricity at a sample rate of 16 kHz for the whole-house and at 1/6 Hz for individual appliances. This is the first open access UK dataset at this temporal resolution. We recorded from five houses, one of which was recorded for 655 days, the longest duration we are aware of for any energy dataset at this sample rate. We also describe the low-cost, open-source, wireless system we built for collecting our dataset.

## Background & Summary

Prudent management of electricity consumption is becoming increasingly important. Yet studies on residential energy users show that the vast majority are poor at estimating their whole-house energy consumption or the energy consumption of individual devices^1^. Residents often underestimate the energy used by heating and overestimate the consumption of perceptually salient devices like lights and televisions^1^. Residents’ failure to correctly estimate energy consumption is likely to lead to higher total consumption.

How significant is occupant behaviour in determining total energy usage? Energy use can differ by two or three times among identical houses with similar appliances occupied by people from similar demographics^2–4^. These large differences in energy consumption are attributed to differences in consumption behaviour. If the house provided better feedback about which devices used the most energy then users could adjust their behaviour to make more efficient use of appliances. ‘Smart electricity meters’ are one such feedback mechanism.

The UK Government requires that energy retailers install smart electricity and gas meters by 2020. The roll-out is already underway^5^. Similar smart meter roll-outs are planned in many countries.

The business case for smart meters in Great Britain^6^ assumes that smart meters will drive savings of £4.6 billion due to reduced energy consumption (across both electricity and gas). Smart meters only provide energy consumption measurements for the entire house yet behavioural research suggests that consumers are best able to manage their electricity consumption when given *appliance-by-appliance* information^7^. Energy disaggregation aims to estimate appliance-by-appliance consumption from a smart meter signal and hence may play an important role in realising the energy savings projected by the smart meter business case.

Energy disaggregation^8^ is an active area of research (see Armel *et al.* ^9^ for a recent review). Researchers require access to large datasets recorded in the field to develop disaggregation algorithms but it is not practical for every researcher to record their own dataset. Hence the creation of open access datasets is key to promote a vibrant research community.

Researchers at MIT led the way by releasing The Reference Energy Disaggregation Data Set (REDD) in 2011 (ref. 10) and more datasets have subsequently been released by researchers in the USA^11–14^, Canada^15^, India^16,17^, France^18^, the UK^19^, Switzerland^20^, Portugal^21^ and Italy and Austria^22^.

To test the performance of a disaggregation algorithm for a specific country, it is important to have access to data from that country because electricity usage varies significantly between countries; both because different countries use different sets of appliances and also because different cultures show different usage patterns.

At the time of writing, the only open-access dataset recorded in the UK is the DECC/DEFRA Household Electricity Study^19^ which has a sample period of two minutes. Yet this sample rate is 12 times slower than UK smart meters which will sample every 10 s^23^. It is this smart meter data which will provide the input to disaggregation algorithms so researchers require access to 10 s data to design disaggregation algorithms for the UK (some other countries will also use smart meters with similar sample periods).

**Figure 1:**
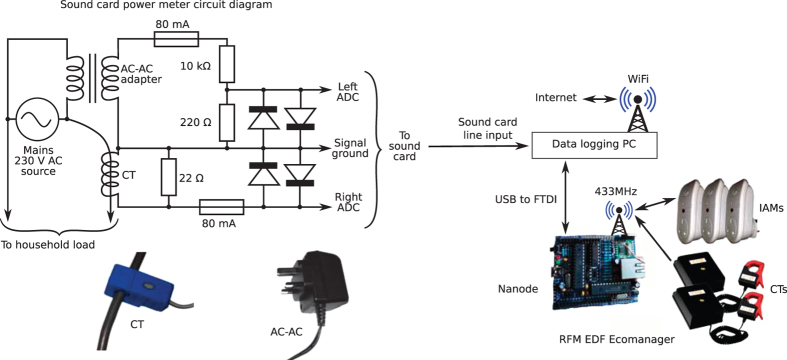
System diagram for the data collection system. The system has three major components: 1) the data logging PC; 2) the sound card power meter and 3) the ‘RFM EDF Ecomanager’ which uses a Nanode to communicate over the air with a set of individual appliance monitors (IAMs) and current transformer (CT) sensors. On the left is the circuit diagram for interfacing a sound card to a CT clamp and AC-AC adaptor to measure mains current and voltage, respectively. The circuit was adapted from Robert Wall's work^31^. Each diode is a 1N5282 (1.3 V forward voltage bias).

We present the first open access UK dataset with a high temporal resolution. We recorded from five houses. Every six seconds we recorded the active power drawn by individual appliances and the whole-house apparent power demand. Additionally, in three houses, we sampled the whole-house voltage and current at 44.1 kHz (down-sampled to 16 kHz for storage) and also calculated the active power, apparent power and RMS voltage at 1 Hz. In House 1, we recorded for 655 days and individually recorded from almost every single appliance in the house resulting in a recording of 54 separate channels (although less channels were recorded towards the start of the dataset). We will continue to record from this house for the foreseeable future. We recorded from the four other houses for several months; each of these houses recorded between 5 to 26 channels of individual appliance data. Figure 1 provides an overview of the system design and Table 1 summarises the dataset.

This dataset may also be of use to researchers working on:

- modelling the electricity grid.
- exploring the potential for automated demand response.
- appliance usage behaviour.

## Methods

Desirable attributes of a dataset for disaggregation include:

- Simultaneously record the power drawn by most of the individual appliances in each house (this data can be used to validate the appliance-by-appliance estimates produced by a disaggregation system or to train the system).
- Record the whole-house active power (this will be the input to the disaggregation algorithm).
- Sample once every 10 s or faster.
- Record for as long as possible.

We first describe our approach to monitoring individual appliances once every 6 s and then describe how we recorded whole-house mains power at 44.1 kHz.

### Individual appliance monitoring

In UK houses such as those in our dataset, mains ‘rings’ extend from the fuse box. Many sockets may share the same ring. Hence, in order to measure individual appliances in the UK, we must install plug-in individual appliance monitors (IAMs) between each appliance and its wall socket.

We used EcoManager Transmitter Plugs^24^ developed by Current Cost and distributed by EDF Energy. The standard base station for these IAMs is the EcoManager^25^. The EcoManager can only handle a maximum of 14 transmitter plugs and only provides data once per minute via its serial port. We needed up to 54 appliances monitored per house and data every 10 s or faster. To achieve this, we set about building our own base station. With the help of others in the community (see Acknowledgements), we reconstructed the specification of the EcoManager protocol.

With the reconstructed protocol in hand, we built our own base station by programming an open-source, rapid-development platform called the Nanode^26^. The Nanode includes an Atmel ATmega328P microcontroller running at 16 MHz (the same microcontroller used on several Arduinos) and a HopeRF RFM12b^27^ radio frequency (RF) module. The EcoManager products appear to use the same (or similar) RF module tuned to the 433 MHz ISM band (note that, in some countries, it is illegal to use the 433 MHz band without a license). We found the appropriate starting point for our RF configuration settings by using a Bus Pirate^28^ to sniff configuration packets from the Serial Peripheral Interface (SPI) connecting the EcoManager’s microcontroller to its RF module.

Each IAM picks its own 32-bit ID at random when the IAM’s ‘pair’ button is pressed. Each IAM stores its ID in non-volatile memory. Our base station maintains a list of these IDs and polls each IAM in order. Each IAM replies to its polling packet within 20 ms, although the power data in this packet may be a few seconds old. The EcoManager RF protocol uses a modular sum checksum byte to provide some resilience against RF corruption. Power data is sent from our Nanode base station to a data logging PC over an FTDI-to-USB cable. It is also possible to turn IAMs on or off remotely.

To measure power from hard-wired appliances such as boilers and kitchen ceiling lights, we used Current Cost transmitters^29^ (TX) with current transformer (CT) clamps. These transmitters use the same radio frequency as the EDF IAMs but a different protocol. In particular, the Current Cost transmitters cannot *receive* RF data. Instead they transmit a data packet once every 6±0.3 seconds without first checking if the RF channel is clear. Hence RF collisions are inevitable and there is no mechanism to request re-transmission of lost data. As such, our base station minimises the chance of packet collisions by learning the transmit period of each Current Cost TX and ensuring that the base station does not transmit for a short window of time prior to the expected arrival of a Current Cost TX packet. Current Cost transmitters do not use a checksum. Instead they use Manchester encoding. RF corruption may result in an invalid Manchester code. If this happens then the receiver can detect the corrupt Manchester code and discard the packet. Unfortunately, it is possible for corruption to damage the data payload without invalidating the Manchester encoding and so corrupt packets are not guaranteed to be detected from Current Cost transmitters.

To maximise the distance over which we can transmit, we experimented with several antenna and RF module configurations. We settled on a quarter-wavelength antenna combined with a ground plane composed of four quarter-wavelength wires in a cross shape running in the plane of the ground, originating from just below the point at which the antenna connects to the Nanode’s printed circuit board.

### Measuring whole-house power demand

Given that there will be a flood of smart meter data in the near future, disaggregation researchers need access to data which is as similar as possible to the data that will be recorded by smart meters in the near future. Unfortunately, ‘real’ smart meters are not trivial to install or to acquire: installation requires an electrician from the utility company and, critically, the current UK smart meter engineering specifications have not yet been finalised (the SMETS2 document^23^ is not an engineering specification). British Gas have installed over a million ‘real’ smart meters but these meters do not comply with SMETS2 and none of our participants had such a meter installed. So we had to build our own measurement system.

The current iteration of the UK smart meter specification^23^ is detailed enough to allow us to build our own metering system which closely mimics what a UK smart meter is likely to provide (these specifications are subject to formal change control processes such that any changes are subject to analysis and stakeholder approval). The latest specifications^23^ state that smart meters are required to connect to the Home Area Network (HAN) using ZigBee Smart Energy Protocol v1. Presumably, a disaggregation system would access smart meter data by way of a ‘Consumer Access Device’ (CAD) connected to the HAN. CADs can request instantaneous active power and pricing data from the smart electricity meter once every 10 s. Additionally, CADs will be able to pull other data such as 13 months of half hourly active import data, 3 months of half hourly reactive import data and 3 months of half hourly active and reactive export data [SMETS2 (ref. 23) and personal communication with DECC, October 2013].

We set out to build a metering system that would collect active power once a second, as well as to sample the voltage and current waveforms at 44.1 kHz to allow researchers who are interested in this high frequency data to use it.

One solution was to use an off-the-shelf Current Cost whole-house transmitter with a current transformer (CT) clamp. These work with our wireless base station. We used this solution in several houses where our bespoke solution was impractical. There are several disadvantages to using a CT clamp connected to a wireless transmitter:

- CT clamps measure current (*I*). The transmitter usually has no way to measure voltage and so must use a hard-coded value for voltage (*V*) to calculate a power reading (*P*) using *P* = *I* × *V*. However, mains voltage in the UK is allowed to vary by +10% to -6% (sometimes quite abruptly) so power readings for a linear resistive load can vary by +20% to -12% (as noted by Hart^8^). These abrupt changes due to external noise are problematic for disaggregation algorithms because disaggregation algorithms tend to rely on *changes* in power demand to detect appliance state changes. However, not all appliances are affected by voltage variations. The power demand for ‘constant power’ devices remains constant across the legal voltage range.
- Battery powered transmitters tend to sparsely sample from their CT clamp in order to minimise battery usage. Hence rapid changes may be missed.
- Without instantaneous measurements of both voltage and current, it is not possible to measure active power or reactive power. Hence CT clamps without voltage measurements can only estimate apparent power.
- The OpenEnergyMonitor emonTx^30^ is unaffected by all three disadvantages mentioned above. However, the version of the emonTx available in 2012 when we designed our system used an analogue to digital converter with only 10 bits of resolution. If we want to measure a primary current which varies from, say, 0 to 30 amps then the emonTx can only resolve changes larger than 14 watts. (The emonTx uses 10 bits of resolution to capture both the positive and negative sides of the AC signal so in effect it uses only 9 bits to cover a 30 amp range. 30A÷2^9^ ADC steps =0.06A per ADC step so it can resolve changes in current larger than or equal to 0.06A. And 0.06A×230V=13.8W). ‘Real’ smart meters will almost certainly have considerably higher resolution so, unfortunately, the emonTx available in 2012 when we were designing our system was not a suitable proxy for a ‘real’ smart meter.

No existing home energy monitor that we were aware of provided an accurate proxy for UK smart meters. Expensive power quality monitors costing several hundred or thousand UK pounds can measure with the accuracy we require but these are prohibitively expensive and some require CT sensors *without* a split core, hence requiring the installer to disconnect the meter tails from the utility company’s meter, which can only be done with permission from the utility company.

We propose a low-cost, high resolution, easy to install technique for recording whole-house mains power demand using a computer sound card, a CT clamp and an AC-AC adapter.

Typical sound cards have remarkably good analogue to digital converters (ADCs). Typical specifications of a modern sound card include:

- 96 kHz sample rate.
- Simultaneous recording of at least 2 channels.
- 90 dB signal to noise.
- 20 bits per sample. Given that each bit provides 6 dB of dynamic range, we effectively have 90/6=15 bits of ‘signal’ and 20−15=5 bits of ‘noise’ per sample.
- Built-in high-pass filter.
- Built-in anti-alias filter.

To record mains voltage and current waveforms we require a simple circuit to connect the sound card to an AC-AC adapter and a CT clamp (Fig. 1). This circuit does not require the user to handle any hazardous voltages. We used the line-input of the sound card rather than the microphone input because the line-input should provide a lower noise signal path than the sound card’s microphone pre-amplifier. The standard maximum peak-to-peak voltage for consumer audio equipment line-input is 0.89 volts. Hence the aim of our circuit must be to reduce the output voltage of each sensor so that we never deliver more than 0.89 volts to the sound card.

To measure mains voltage as safely as possible, we used a standard AC-AC adapter (the ‘Ideal Power 77DB-06-09’). This provides a peak open-circuit output voltage of approximately 11 volts. Research done by the Open Energy Monitor project^31^ suggests that the output of the AC-AC adapter should track the mains input voltage linearly over the range 185.5 to 253 V. We reduced the AC-AC adapter’s output voltage with a voltage divider circuit (we used two resistors: 10 kΩ and 220 Ω) to produce about 0.7 V peak-to-peak which is fed into one channel of the sound card’s line input.

To measure mains current, we used a current transformer (CT) clamp (the ‘YHDC SCT-013-000’). Both the CT clamp and AC-AC adapter were sourced from the Open Energy Monitor shop^32^. The CT is connected in parallel to a 22 Ω burden resistor. This configuration produces about 0.89 V peak-to-peak across the burden resistor when the CT is presented with a primary current of 30 amps RMS which, we believe, is the most current that any of the houses under study will pull.

To protect the sound card against overload, both channels include an 80 mA quick-blow fuse and a pair of 1N5282 diodes (with a 1.3 V forward voltage bias) to ensure that the circuit is unlikely to ever deliver more than 1.3 V to the sound card.

Let us calculate a rough estimate for our measurement resolution. If we want to measure a primary current with a range of 0 to 30A_rms_ then we should be able to resolve changes in primary current of approximately 3mA per sample ( ${30A}_{\mathrm{rms}}\times \sqrt{2}\times 2\approx {85A}_{\mathrm{peak}‐\mathrm{to}‐\mathrm{peak}}$${85A}_{\mathrm{peak}‐\mathrm{to}‐\mathrm{peak}}$÷2^15^ADC steps≈3mA). For the voltage measurement, if we want a range of 0 to 253V_rms_ (230V_rms_ + 10%) then we should be able to resolve changes of approximately 22mV per sample ($253{\mathrm{volt}}_{\mathrm{rms}}\times \sqrt{2}\times 2\approx {716V}_{\mathrm{peak}‐\mathrm{to}‐\mathrm{peak}}$${716V}_{\mathrm{peak}‐\mathrm{to}‐\mathrm{peak}}$$2^{15}\mathrm{ADC}\;\mathrm{steps}\approx 22mV$). Given that the sensors are likely to be noisy and given that we are only providing 0.7V_peak‐to‐peak_ to the ADC for the voltage measurements, we should downgrade our resolution per sample to about 30mV and 5mA for voltage and current respectively. This gives us a resolution for power of approximately 30mV×5mA=150mW.

We now describe the software for our sound card power meter. We use the following relations to calculate |*S*| (apparent power) and *P* (‘real’ or ‘active’ power) from simultaneously recorded vectors of voltage and current readings (we record in chunks each with a duration of 1 s; this time period was chosen because REDD uses this sample period for mains data):

(1)|S|=Irms×Vrms

(2)P=1N∑i=1NIiVi

Where *I*_rms_ and *V*_rms_ are the root mean squared values for the current and voltage vectors respectively; *N* is the number of samples; *I*_*i*_ and *V*_*i*_ are the *i*th samples of the current and voltage vectors respectively. The system does not guarantee that we always process chunks of length equal to precise integer multiples of the mains cycle period but, as demonstrated in the Technical Validation section, we still achieve relative errors consistently less than 2%.

The conclusion is that we achieve a resolution greater than that required to provide a good proxy for ‘real’ smart meters (although we acknowledge that we do not know the precise resolution of ‘real’ smart meters. This decision is likely to be left to the manufacturers [personal communication with DECC, March 2013]). We save *P*, |*S*| and *V*_*rms*_ to disk once a second with a precision of 2 decimal places in a CSV file.

We also save the raw ADC data to disk. To reduce the space required, the ADC data are down-sampled using the open-source audio tool sox^33^ to 16 kHz (REDD used 15 kHz and we originally wanted to use the standard defined by REDD but we found that support for 16 kHz is more common than for 15 kHz in processing tools). The ADC is 20-bit but few audio processing tools can process 20-bit files so we pad each sample to produce a 24-bit file. The uncompressed 16 kHz 24-bit files would require 28.8 GBytes per day so we compress the files using the Free Lossless Audio Codec (FLAC)^34^ to reduce the storage requirements to ≈ 4.8 GBytes per day.

#### Calibration

To convert the raw ADC values to voltage and current readings, we must first find appropriate conversion constants. We calibrate each data collection system separately to compensate for manufacturing variability in the components. We calibrate each system once when it is first setup. We connect a ‘Watts up? PRO meter^35^’ to the data logging PC via USB during setup to automatically calibrate voltage and current conversion factors. We typically use a resistive load like a kettle to calibrate the system. If the ‘Watts up?’ meter reports a power factor greater than 0.97 then the calibration script also calibrates the phase shift introduced by the sensors.

#### Open source implementation

We have implemented the power monitoring system described as five software projects. All software packages are available from https://github.com/JackKelly/<package name>. The packages are:

**rfm_edf_ecomanage**r:

Nanode C++ code. This code allows the Nanode to talk directly to multiple Current Cost whole-house sensors (CC TXs) as well as to multiple EDF Transmitter Plugs (CC TRXs). Users talk to the Nanode over the serial port. Users send simple commands. It sends data back to the PC in a simple JSON format.

**rfm_ecomanager_logger:**

A Python script for communicating with the rfm_edf_ecomanager Nanode system. This provides a command-line tool for ‘pairing’ sensors with the logging system; assigning human-readable names to those sensors and then recording the data to disk in CSV files using the same format as MIT’s REDD files. The emphasis is on reliable logging. rfm_ecomanager_logger attempts to restart the Nanode if the Nanode crashes. rfm_ecomanager_logger ensures, as far as possible, that time stamps are correct (which is not trivial given that the Nanode does not have a real time clock and given that serial data could be kept in the operating system’s buffer if the system is under heavy load). Data are recorded approximately once every six seconds for each channel.

**powerstats:**

Produce statistics and graphs from REDD-formatted power data. Mainly used for checking the health of sensors.

**babysitter:**

A Python module for ‘babysitting’ each logging system. Sends an email if a sensor stops working or if rfm_ecomanager_logger fails. Also sends a ‘heartbeat’ email once a day to the home owner containing statistics (created by powerstats) describing the last day’s power data. Also provides useful ‘health’ information about the system such as remaining disk space.

**snd_card_power_meter:**

System for recording voltage and current waveforms at 44.1 kHz, 20-bit per channel using a PC’s sound card. Calculates and saves active power, apparent power and RMS voltage to a CSV file once a second. Records down-sampled ADC data to a FLAC file.

### Complete metering setup

To collect our own dataset, we installed the following equipment in each house:

- Multiple EDF Individual Appliance Monitors.
- A CurrentCost CT clamp and transmitter to measure whole-house apparent power. House 1 used additional CC CT clamps to measure the lighting circuit, kitchen ceiling lights, boiler and solar hot water pump.
- Nanode running our rfm_edf_ecomanager code.
- A small-footprint Atom PC (full component listing of the Atom PCs we built can be found at jack-kelly.com/intel_atom_notes and a guide to setting up a complete data logging system can be found at github.com/JackKelly/rfm_ecomanager_logger/wiki/Build-a-complete-logging-system). We used the Intel DN2800MT motherboard (with a Realtek ALC888S audio codec capable of sampling at 96 kHz 20-bit resolution with a signal to noise ratio of 90 dB; and a line-input socket on the rear) and a 320 GB HDD; runs Ubuntu Linux Server; consumes 14 W active power.
- Houses 1, 2 and 5 had the sound card power meter system installed to measure whole-house active and reactive power and voltage.

A system diagram is shown in Fig. 1.

CSV data files recorded by the data logging PC were transmitted to a remote server every morning using rsync^36^. FLAC files were transferred manually using an external hard disk every two months.

#### Known issues

- Each IAM draws a little power (active power ≈ 0.9 W; apparent power ≈ 2.4 VA). House 1 has IAMs installed on almost every appliance and the correlation of the sum of all submeters in House 1 with the mains is 0.96. Yet the proportion of energy submetered in House 1 is only 80%. This reasonably low value for the proportion of energy submetered is likely due in large part to the fact that the 52 EDF IAMs installed in House 1 draw approximately 50 W, yet this power is not measured by the individual appliance meters.
- The IAMs and the Current Cost transmitters occasionally report spurious readings. rfm_ecomanager_logger filters out any readings above 4 kW for IAMs and above 20 kW for whole-house readings. 4 kW is above the safe maximum power draw for a UK mains appliance (2.99 kW=13 amps ×230 volts). 20 kW is more than twice the maximum whole house power reading we have recorded (8.765 kW) across all houses in our dataset.
- Whilst the Atom motherboard’s ADC is capable of sampling at 96 kHz, we had to use 44.1 kHz because the system produced buffer overflow errors if the sample rate was above 44.1 kHz. There remain some missing samples in the 16 kHz data due to buffer overflow errors during recording.

### Selecting houses to record

The subjects were either MSc students or PhD students at Imperial College. The subjects chose to do a research project with the authors. To assist in both their own project and in the collection of the UK-DALE dataset, the students kindly agreed to install metering hardware in their house. The upper bound on the number of houses we could record from was set by a combination of a limited financial budget, limited time to assemble the metering hardware, and a limit in the number of students who volunteered to work on research projects relating to domestic energy consumption.

Within each house, the home owner selected which appliances to record, with the recommendation from the authors that the most energy hungry appliances should take priority.

## Data Records

UK-DALE uses a data format similar to that used by the first public disaggregation dataset, the Reference Energy Disaggregation Data Set (REDD)^10^.

There are five directories, one per house. The directories are named house_<x> where *x* is an integer between 1 and 5.

Each directory contains a set of channel_<i>.dat CSV files (one file per electricity meter *i*) and a labels.dat file which is a CSV file which maps from channel number *i* to appliance name. All CSV files in UK-DALE use a single space as the column separator (as per REDD).

One way in which UK-DALE differs from REDD is that UK-DALE includes a set of detailed metadata files. These follow the NILM Metadata schema^37^. The metadata files are in YAML text file format (YAML is a superset of the JSON format). This metadata describes properties such as the specifications of each appliance; the mains wiring between the meters and between meters and appliances; exactly which measurements are provided by each meter; which room each appliance belongs in etc. The labels.dat file in each directory is redundant and is only included to provide compatibility with REDD.

All data in UK-DALE as of January 2015 are available from the UK Energy Research Council’s Energy Data Centre. The data are also available from www.doc.ic.ac.uk/~dk3810/data/. The latter source will be updated as we collect more data. There are three forms of data in UK-DALE:

- The 6 second data from the Current Cost meters (Data Citation 1).
- The 1 second data from our sound card power meter (Data Citation 1).
- The 16 kHz data recorded by our sound card power meter (Data Citation 2). The complete set of 16 kHz files requires 4 TBytes of storage. The 16 kHz data is supplied as a set of 200 MByte files. Each file records 1 h of data.

The 6 s data and 1 s data are stored in CSV files, one CSV file per meter. The first column is a UNIX timestamp (the number of seconds elapsed since 1970-01-01 00:00:00 UTC). The UNIX timestamp is UTC (Coordinated Universal Time) and hence ignores daylight saving transitions (the UK is UTC+0 during winter and UTC+1 during summer).

The 1 s data, 6 s data and metadata are also available as a single HDF5 binary file, ready for use with the open-source energy disaggregation toolkit NILMTK^38^. The UK-DALE HDF5 binary file is available from www.doc.ic.ac.uk/~dk3810/data.

### 6 second data

For the 6 s data, the second column in each CSV file is a non-negative integer which records power demand of the downstream electrical load. The file names of the 6 s data take the form of channel_<X>.dat where X is a positive integer (no leading zero). There are two types of 6 s resolution meters:

1. Individual appliance monitor transmitter plugs that record *active* power (in units of watts).
2. Current transformer meters that record *apparent* power (in units of volt-amperes).

Individual appliance monitors have a push-button switch to allow users to turn the connected appliance on and off. We record the activity of this switch in a channel_<X>_button_press.dat file. If the switch has just been toggled *on* then a ‘1’ is recorded. If the switch has just been toggled *off* then a ‘0’ is recorded. The motivation behind logging switch press events is that these provide (imperfect) room occupancy information.

Switch *on* events should be a perfectly clean recording (i.e. the only possible reason for an on-switch event appearing in the data is that the user pressed the switch). Unfortunately, off-switch events may include false positives. Occasionally IAMs turn off spontaneously (an event which is impossible to distinguish from a genuine button press). Also, if power is lost and returned to the IAM within 12 s then this will be logged as an off-switch event. If the power is off for more than 12 s then the system assumes that the IAM was deliberately unplugged and hence the system will switch the IAM to its previous power state when it reappears; this automatic switch event is not recorded.

### 1 second data

There are four columns in each CSV file recording the whole-house power demand every second:

1. UNIX timestamp.
2. Active power (watts).
3. Apparent power (volt-amperes).
4. Mains RMS voltage.

All four columns record real numbers (not integers). The first column has one decimal place of precision; the other columns have two decimal places of precision. The 1 s data is in a CSV file called mains.dat in directories house_1, house_2 and house_5.

### 16 kHz data

The 16 kHz data is compressed using the Free Lossless Audio Codec (FLAC)^34^. For houses 1, 2, and 5 UK-DALE records a stereo 16 kHz audio file of the whole-house current and voltage waveforms. The files are labelled vi-<T>.flac where *T* is a real number recording the UNIX timestamp with micro-second precision (using an underscore as the decimal place). This timestamp is the time at which the audio file began recording. The recordings are split into hour-sized chunks. We also include a calibration.dat file for each house. This is a text file specifying the multipliers required to convert the raw output of the analogue to digital converter to amps and volts.

To make use of the FLAC files (for processing in, for example, MATLAB or Python), first decompress the files to create WAV files. This decompression can be done with many audio tools. We use the audio tool sox
^33^.

With the WAV files in hand, the next task is to convert from the values in the WAV files (in the range [−1,1]) to volts and amps. Use the calibration.cfg file for the house in question. This file specifies an amps_per_adc_step parameter and a volts_per_adc_step parameter. Users can safely ignore the phase_difference parameter and assume that the measurement hardware introduces no significant phase shift. Use the following formula to calculate volts from the WAV files:

$$\mathrm{volts}=\mathrm{value}\;\mathrm{from}\;\mathrm{WAV}\times \mathrm{volts}\;\mathrm{per}\;\mathrm{ADC}\;\mathrm{step}\times 2^{31}\;\mathrm{ADC}\;\mathrm{steps}$$

Use a similar formula for amps. To explain the formula above: The recording software stores each sample as a 32 bit integer. Hence there are 2^32^ ADC steps for the full range from [−1,1] and 2^31^ ADC steps for half the range.

## Technical Validation

Table 1 summarises the UK-DALE dataset. The table includes some metadata (which is also recorded in the machine-readable metadata supplied with the dataset) including the type of building, the year of construction, the main heat source, whether the property is bought or rented, the number of occupants, a description of the occupants, the total number of meters, the number of site meters, the sample rate of the mains meters and the start and end dates for the recordings. The table also includes summary statistics calculated using the open source energy disaggregation tool NILMTK^38^: the average mains energy consumed per day, the correlation of the mains meter with the sum of all submeters, the proportion of energy submetered, and the dropout rate. The values for the average energy consumption per day are close to the value of 9.97 kWh per day reported in DECC’s Household Electricity Survey^19^ (which surveyed 251 houses in the UK), hence we can have some confidence that our houses consumed a fairly typical amount of energy for a UK house.

**Figure 2:**
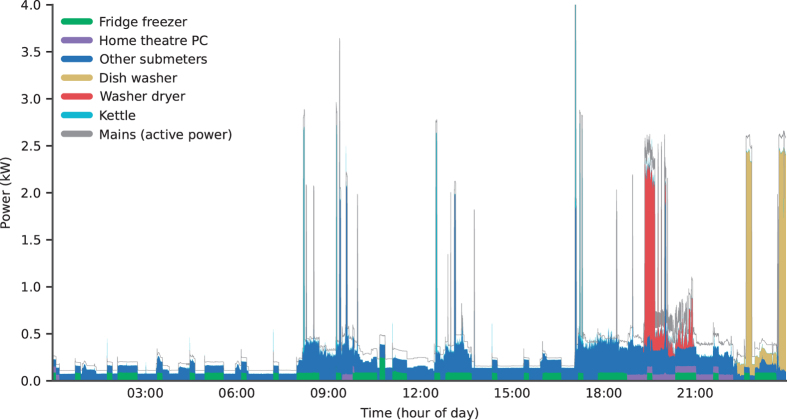
Power demand for a typical day (Sunday 2014-12-07) in House 1. The thin grey line shows the mains (whole-house) active power demand recorded using our sound card power meter. The stacked and filled coloured blocks show the power demand for the top five appliances (by energy consumption) and the dark blue block shows all the other submeters summed together. The thin white gap between the top of the coloured blocks and the mains plot line represents the power demand not captured by any submeter.

**Figure 3:**
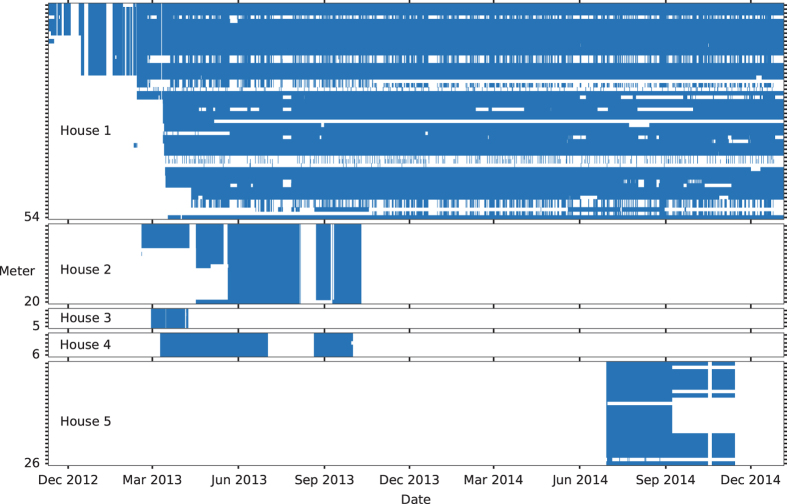
Time periods when meters were recording. The five houses in the dataset are represented by the five panels in this plot. The height of each panel is proportional to the number of meters installed in each house. Each thin row (marked by each y-axis tick mark) represents a meter. Blue areas indicate time periods when a meter was recording. White gaps indicate gaps in the dataset.

**Figure 4:**
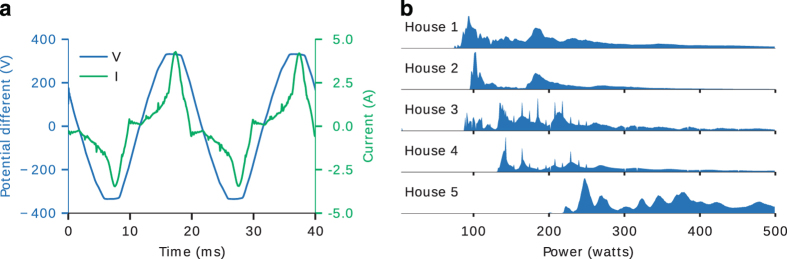
Mains electricity data. (**a**) 16 kHz sampling of mains voltage and current using our sound card power meter from House 1 on 2014-09-03 21:00:00+01:00. The green line shows the current and the blue line shows the voltage. (**b**) shows histograms of mains power demand for each house. The five subplots represent the five houses in the dataset. There is some density above 500 watts but this has been cropped from this plot to allow us to see detail in the range between 0 and 500 watts.

**Figure 5:**
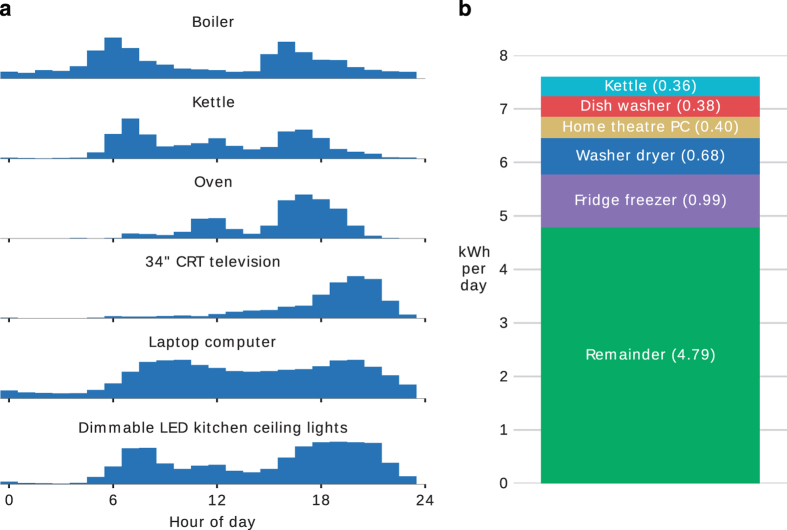
Electrical appliance usage in House 1. (**a**) Histograms of daily appliance usage patterns. (**b**) shows average daily energy consumption of the top-five appliances in House 1. All appliances were ranked by the amount of energy they consumed and the top-five are shown here. All lights were grouped together. The ‘remainder’ block at the bottom represents the difference between the total mains energy consumption and the sum of the energy consumption of the top five appliances. As such, the top edge of the bar shows the average daily total energy consumption for House 1.

**Figure 6:**
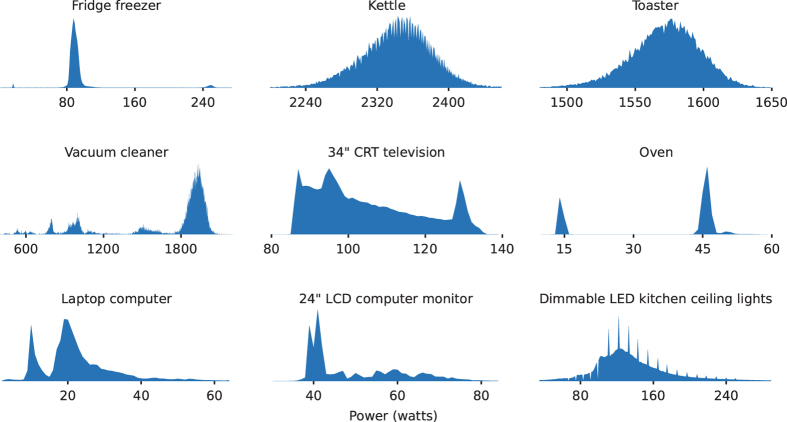
Histograms of appliance power demand from House 1.

Figures 2, 3, 4, 5, 6 (except panel ‘a’ in Fig. 4) were produced using NILMTK. The scripts to generate these plots are available at github.com/JackKelly/ukdale_plots.

Figure 2 shows the power demand for a typical day for House 1. We show the individual power demand for the top-five appliances (ranked by energy consumption) and all other submeters summed together. We also show the whole-house mains power demand. The difference between the mains power demand and the top of the submetered power demand illustrates the small amount of energy which is not submetered.

The time periods when each meter was capturing data is shown in Fig. 3. Note that the numerous gaps in the data from House 1 are almost all deliberate and not the result of an equipment failure. For example, some meters in House 1 are manually turned off if the attached appliance is unplugged.

An example of 16 kHz data captured by our sound card power meter is shown in Fig. 4 panel ‘a’. Note that the voltage is almost a pure 50 Hz sine wave but the current contains many harmonics.

The distribution of values for the mains power demand for each house is shown in Fig. 4 panel ‘b’. The left-most edge of each density represents the ‘vampire power’ of each house (i.e. the power demand when no one is using an appliance but power is still being drawn by always-on appliances and appliances in standby mode).

Figure 5 panel ‘a’ shows the hour per day that several appliances are used. For example, the oven shows two peaks in usage: one around midday (lunch) and one around 18:00 (dinner).

The energy consumed by the top five energy consuming appliances in House 1 is shown in Fig. 5 panel ‘b’. This is relevant because energy disaggregation researchers often prioritise the disaggregation of the appliances responsible for the largest energy consumption.

The distribution of values of power demand for individual appliances is shown in Fig. 6. Some appliance information can be inferred from the histograms. For example, the top left panel shows a histogram for the power demand of the fridge: the main peak around 90 W is the normal compressor cycle, the peak around 17 W is the fridge lamp and the peak around 250 W is the defrosting cycle. The vacuum cleaner has six discrete power settings, all of which can be seen in its histogram.

**Figure 7:**
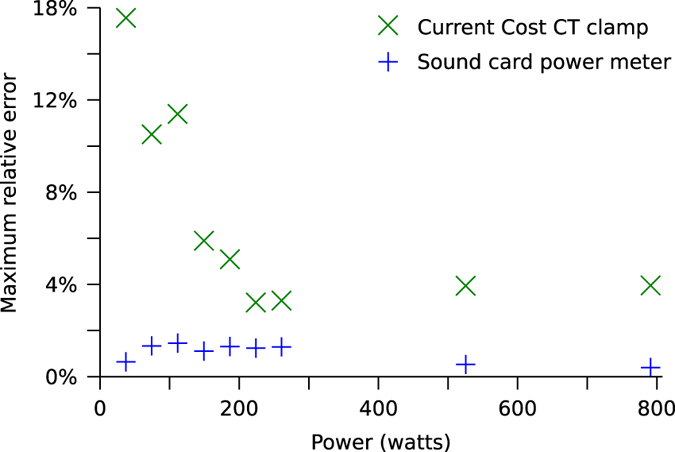
Maximum relative measurement error for power measurements across a range of loads. A `Watts up? PRO' meter^35^ was used to record the ground-truth.

Figure 7 shows the measurement errors for our sound card power meter and the Current Cost Current Transformer (CT) sensor across a range of resistive loads. The ground truth was measured using a ‘Watts up? PRO’ meter^35^ (with a stated accuracy of ±1.5%). The range of loads were created by using three incandescent lamps and by changing the number of primary turns on the CT from one to seven, in steps of one. For each load, we recorded one minute of data and took the largest error from that minute of data. The sound card power meter was calibrated six months prior to the test. This illustrates that our sound card power meter consistently produces a relative error of less than 2% and that the Current Cost CT meter produces errors of less than 6% as long as the power is above 100 watts (the whole-house power demand very rarely drops below 100 watts).

We compared the total active energy recorded by our sound card power meter for House 1 with the utility-installed ‘spinning disk’ electricity meter in House 1. We selected the time period of 2014-11-28 08:55 to 2013-05-22 20:36 for the comparison because we have a continuous recording from our sound card power meter for this period, and we have readings of the utility meter at the start and end of this period. The total energy recorded by the utility meter for this period was 4030.60 kWh. The total recorded by the sound card power meter was 4142.93 kWh. The relative difference is 2.71%.

Houses 1, 2 and 5 had two mains meters: a sound card power meter and a Current Cost meter. For each of these houses, we compared the apparent energy recorded by the sound card power meter against the Current Cost whole-house meter. The relative difference was 1.79% in House 1; 7.30% in house 2 and 5.68% in house 5.

## Usage Notes

Any software designed to process REDD files should be able to open UK-DALE data (although the extra metadata provided by UK-DALE will be ignored).

The open-source energy disaggregation toolkit NILMTK^38^ includes an importer for UK-DALE data. NILMTK can handle the metadata provided with UK-DALE. An HDF5 version of the UK-DALE dataset (for use with NILMTK) is available for download (please see the Data Records section of this paper).

There are several aspects of the dataset that might need to be addressed using appropriate pre-processing:

Data packets from the wireless meters are occasionally lost in transmission. Around 6% of packets are lost from the Current Transformer sensors and around 0.02% of packets are lost from Individual Appliance Monitor plugs. The sample period for the 6 s data may drift up or down by a second.

Some individual appliance monitors are switched off (and hence do not transmit any data) when the appliance is switched off from the mains. We switch them off to reduce the risk of an electrical fault causing a fire, and to save energy. Some appliances are unplugged for the majority of the time. For example, the vacuum cleaner is physically unplugged from the wall socket when not in use and the vacuum cleaner’s meter is left attached to the cleaner’s power plug (and hence is not powered). As a rule of thumb, any gap in the data longer than two minutes can be assumed to be caused by the appliance (and monitor) being switched off from the mains. Hence gaps longer than two minutes can safely be filled with zeros. The threshold of two minutes was chosen because we observed gaps less than two minutes caused by a succession of radio transmission errors. Any gap shorter than two minutes can be forward-filled from the previous reading.

Some appliances draw more than 0 watts when nominally ‘off’. We typically use 5 watts as the threshold between ‘on’ and ‘off’. The metadata includes an on_power_threshold property for each appliance. This property is present if the on power threshold for that appliance is not 5 watts.

NILMTK contains preprocessing tools for handling these scenarios.

## Additional information

**How to cite this article:** Kelly, J. and Knottenbelt, W. The UK-DALE dataset, domestic appliance-level electricity demand and whole-house demand from five UK homes. *Sci. Data* 2:150007 doi: 10.1038/sdata.2015.7 (2015).

## Supplementary Material



## Figures and Tables

**Table 1:** Summary statistics for each house.

House	1	2	3	4	5
Building type	end of terrace	end of terrace		mid-terrace	flat
Year of construction	1905	1900		1935	2009
Energy improvements	solar thermal & loft insulation & solid wall insulation & double glazing	cavity wall insulation & double glazing		loft insulation & double glazing	
Heating	natural gas	natural gas		natural gas	natural gas
Ownership	bought	bought		bought	bought
Number of occupants	4	2		2	2
Description of occupants	2 adults and 1 dog started living in the house in 2006. One child born in 2011. Second child born in 2014.	2 adults. 1 at work all day; the other sometimes home		1 adult and 1 pensioner	2 adults
Total number of meters	54	20	5	6	26
Number of site (mains) meters	2	2	1	1	2
Sample rate of mains meters	16 kHz & 1 Hz & 6 s	16 kHz & 1 Hz & 6 s	6 s	6 s	16 kHz & 1 Hz & 6 s
Date of first measurement	2012-11-09	2013-02-17	2013-02-27	2013-03-09	2014-06-29
Date finished installing all meters	2013-04-12	2013-05-22			
Date of last measurement	2015-01-05	2013-10-10	2013-04-08	2013-10-01	2014-11-13
Date when some meters were removed					2014-09-06
Total duration (days)	786	234	39	205	137
Total uptime for mains meter (days)	655	140	36	155	131
Uptime proportion	0.83	0.60	0.93	0.75	0.96
Average mains energy consumption per day (active kWh)	7.64	7.17			13.75
Average mains energy consumption per day (apparent kVAh)	8.90	8.00	12.35	10.24	17.56
Following statistics calculated when all meters installed					
Correlation of sum of submeters with mains	0.96	0.86	0.47	0.55	0.90
Proportion of energy submetered	0.80	0.68	0.19	0.28	0.79
Mean dropout rate (ignoring large gaps)	0.02	0.02	0.02	0.02	0.02
‘uptime’ is the total time that the system was active and recording. The ‘total duration’ is ‘date of last measurement’ minus ‘date of first measurement’. The correlation of the mains meter with the sum of all submeters gives an indication of how much of the variance in the mains signal is captured by the submeters. The proportion of energy submetered is the total energy captured by the submeters divided by the total energy captured by the mains meter. The dropout rate (ignoring large gaps) gives a measure of the rate at which packets were lost due to radio errors (large gaps are ignored because these are often caused by a meter being deliberately unplugged). Some metadata is not available because the occupants are no longer contactable.					
